# Bioactive compounds, pharmacological actions and pharmacokinetics of *Cupressus sempervirens*

**DOI:** 10.1007/s00210-022-02326-z

**Published:** 2022-11-17

**Authors:** Gaber El-Saber Batiha, John Oluwafemi Teibo, Hazem M. Shaheen, Opeyemi Abigail Akinfe, Aya Ahmed Awad, Titilade Kehinde Ayandeyi Teibo, Athanasios Alexiou, Marios Papadakis

**Affiliations:** 1 Department of Pharmacology and Therapeutics, Faculty of Veterinary Medicine, Damanhour University, Damanhour, 22511 AlBeheira Egypt; 2 Department of Biochemistry and Immunology, Ribeirão Preto Medical School, University of São Paulo, Ribeirão Preto, São Paulo Brazil; 3 Department of Biochemistry, University of Ibadan, Ibadan, Nigeria; 4 Department of Maternal-Infant and Public Health Nursing, College of Nursing, University of São Paulo, Ribeirão PretoRibeirão Preto, SP Brazil; 5 Department of Science and Engineering, Novel Global Community Educational Foundation, Hebersham, NSW 2770 Australia; 6 AFNP Med, 1030 Wien, Austria; 7 Department of Surgery II, University Hospital Witten-Herdecke, University of Witten-Herdecke, Heusnerstrasse 40, 42283 Wuppertal, Germany

**Keywords:** *Cupressus sempervirens*, Biological properties, Chemical analysis, Conventional uses, Ethnomedicine, Interactions, Pharmacological activities, Phytochemicals

## Abstract

The roles of plants and its products in all forms of life cannot be overemphasized. The medicinal products from plant are phytochemicals, drugs, food supplements, beauty products, etc. In ethnomedicine, leaves, fruits, stem, bark, root and fluids from plants are used in the cure, management and prevention of several diseases. *Cupressus sempervirens*, sometimes called Italian or Mediterranean cypress, is found in subtropical Asia, North America and eastern Mediterranean region. Pharmacological investigations of *Cupressus sempervirens* showed biological properties such as aromatherapeutic, antiseptic, astringent, balsamic or anti-inflammatory, astringent, antiperspirant, diuretic and antispasmodic. Chemical analysis of *Cupressus sempervirens* gives phytochemicals like monoterpenes, diterpenes, flavonoid glycosides and bioflavonoids. The current review highlights interactions, conventional uses and biological actions of *Cupressus sempervirens* plant and plant products.

## Introduction

**Fig. 1:**
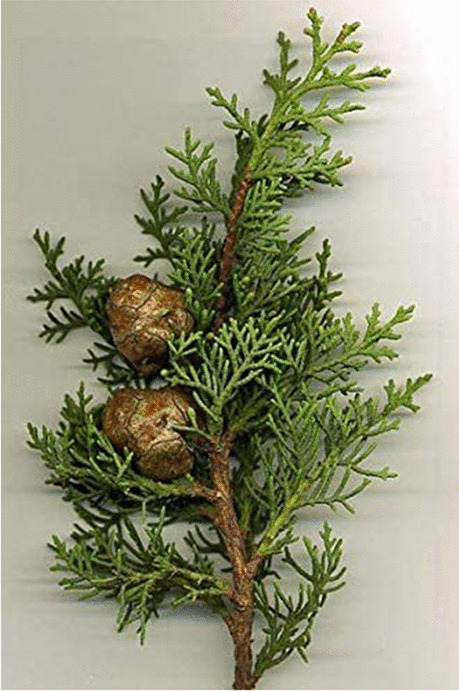
Cupressus sempervirens

In the last two decades, the need to research medicinal plants has expanded enormously around the world. In 1978, World Health Organization emphasized the importance of scientific research into herbal medicine. Herbs are generally valued for their virtues as food as well as medicine (Oliver 1960, Balogun et al. 2016). From the evidence gathered, medicinal plants are source of compounds which are free radical scavengers and antioxidants that may mitigate oxidative stress–induced pathologies and toxicities (Rossato et al. 1999; Famurewa et al. 2018). Parts of plants produce various secondary metabolites added as formulations in pharmaceutical drugs. Plants have the ability to synthesize a wide variety of chemical compounds that perform important biological functions to protect against attack from microorganisms (Al-Snafi 2015a). The wide scope of the pharmacological and therapeutic effects of medicinal plants has been shown in several reviews. About 270,000 species of flowering plants has been identified; only 0.5% have details on chemical composition and medicinal value. In fact, researchers have less than 5% information on the chemical composition of the flowering plants in the rainforest (Jackson 1989; Al-Snafi 2015b; Balogun et al. 2016). The study of traditional uses of plants is recognized as an effective way to discover future medicines. The use of these plants in medicine is due to the presence of bioactive constituents such as phenols, flavonoids, tannins and alkaloids, present either in the seeds, leaves, stems or roots (Tapsell et al. 2006, Balogun et al. 2016). Preliminary phytochemical analyses of *Cupressus sempervirens* revealed that essential oils, alkaloids 0.7%, saponins 1.9%, flavonoids 0.22%, phenols 0.067%, tannins 0.31%, and other bioactive compounds were present in the plant. Medical examination in the past found that *Cupressus sempervirens* contained antioxidant, anticancer, antifungal, antibacterial, antiparasitic, antiviral, insecticidal, anticoagulant, estrogenic, healing and numerous properties (Fig. 1).

## Synonyms and common names

*Cupressus sempervirens L*. subspecies horizontalis (Mill.) A. Camus, *Cupressus sempervirens L*. variety sphaerocarps (Parl.) Parl., *Cupressus sempervirens L*. variety umbilicata (Parl.) Parl., *Cupressus sempervirens L*. Forma stricta (Aiton) Rehder, *Cupressus sempervirens L.* subspecies indica (Parl.) Silba, *Cupressus sempervirens L.* variety Atlantic (Gaussen) Silba, *Cupressus sempervirens L.* variety dupreziana (Camus) Silba, *Cupressus sempervirens L.* variety globulifera Parl., *Cupressus sempervirens L.* variety horizontalis (Mill) Loudon, *Cupressus sempervirens L.* variety indica Parl., *Cupressus sempervirens L.* variety numidica Trab., *Cupressus sempervirens L.* variety pendula (Endl.) A. Camus, *Cupressus sempervirens L.* variety stricta Aiton (Farjon 2013, Al-Snafi 2016). Arabic: Saro, Shajarat el-Saro, Saro al-bahr al-abiadh; Chinese: say zhong hai bai mu; English: common cypress, cemetery cypress, Italian cypress, Mediterranean cypress, Tuscan cypress, pencil pine; French: *Cyprès commun*, *Cyprès de Montpellier, Cyprès de Provence, Cyprès d'Italie, Cyprès mediciterranéen, Cyprès ordinaire, Cyprès pyramidal, Cyprès sempervirent, Cyprès toujours vert*; Italian: commune of Cipresso; Spanish: Common cypress, Italian cypress; Swedish: kretacypress German: *Echte Zypresse, Italienische Zypresse* (Chaudhary et al. 2012).

**Taxonomic classification** and distribution.

*C. sempervirens* was local to the Mediterranean basin, though, it originates from continents like Asia, North Africa, Southern Europe and North America (Fleming 2000, Chaudhary et al. 2012) (Table 1).

**Table 1:** Taxonomic classification of *C. sempervirens*

Kingdom	Plantae
Subkingdom	Viridiplantae
Infrakingdom	Streptophyta
Superdivision	Embryophyta
Division	Tracheophyte
Subdivision	Spermatophytin
Class	Pinopsida
Subclass	Pinidae
Order	Pinales
Family	Cupressaceae
Gender	Cupressus
Species	*Cupressus sempervirens*

## Essential oil chemical compositions

In 2010, Mazari et al. investigated essential oil leaves extraction of *C. sempervirens* and *Juniperus phoenicea* by steam distillation, of which 0.52% and 0.26% (w / w and w / p) from *J. phoenicea* and *C. sempervirens* were obtained respectively (Mazari et al. 2010). Studies on *J. phoenicea* obtained from Morocco have been able in the same line to obtain a yield of 1.62, 0.94 and 0.70% (p/p) of Moroccan leaves (Barrero et al. 2006; Achaket al. 2009; Derwich et al. 2010). *C. sempervirens* obtained from Cameroon has lower yield up till thrice compared to that from Algeria (1%) (w / w)(Tapondjouet al. 2005). The analysis of *J. phoenicea* and *C. sempervirens* essential oil using GC–MS is presented in Table 2 showing 35–36 compounds (91.6–96.9%) respectively, while the oil from Morocco and Tunisia has 45 (72%) and 31 (99%) identified compounds (Achak et al. 2008). The oils are made up of monoterpenic hydrocarbons (72.9–75.7%), α-pinene (34.5–60.5%). *J. phoenicea* oil has Β-phelandrene (22.4%) and α-terpinyl acetate (14.7%) as its second most important constituents (Mazari et al. 2010). But for *C. sempervirens* oil cedrol (8.3%) is the second most important component; this is similar to Moroccan *J. phoenicea* (Barrerol et al. 2006; Achak et al. 2008, 2009). The commonest component of essential oils are monoterpenes (71.1%), α-pinene (45.5%) and 38.2–58% (Achak et al. 2009), but in Moroccan leaf essential oil, δ-3-carene (13.0%) (Barrero et al. 2006) and 7.6% (Achak et al. 2009), but Algerian leaves have about 4.7%. Similar report was given by Rezzi et al. (2001) and Cavaleiro et al. (2001) showing α-pinene, β-phelandrene and α-terpinyl acetate as the fundamental components of the essential oil of leaves gotten from the Portuguese and Corsican *J. phoenicea* (Cavaleiro et al. 2001; Rezzi et al. 2001). In the case of *C. sempervirens*, Tognolini et al. (2006), α-pinene is the main component of the essential oil of leaves, but α-pinene is presented in less content (26.4%) compared to Mazari group’s study (60.5%) (Mazari et al. 2010), while α-pinene is the second and third significant component, respectively, in previous research (Sacchetti et al. 2005; Tapondjouet al. 2005) (Table 3).

**Table 2:** Chemical composition of essential oils from and *C. sempervirens* (Mazari et al. 2010; Rguez et al. 2022)

Compound	*C. sempervirens* (%)	
Tricycle	0.5	
α-Thujene	0.4	
α-pinene	60.5	
Camphene	0.5	
Sabinene	1.3	
β-pinene	2.9	
Myrcene	3.9	
α-phelandrene	-	
δ-3-carene	0.2	
α-terpinene	0.2	
p-cimeno	0.2	
Limonene	4.6	
β-phelandrene	-	
γ-terpinene	0.5	
Terpinolene	two	
Linalol	tr	
Pinocarveol	tr	
Borneol	tr	
α-terpineol	tr	
Citronellol	-	
Thymol methyl ether	0.2	
Linalyl acetate	tr	
Bornyl acetate	0.3	
Carvacrol	tr	
Thymol	tr	
α-terpinyl acetate	-	
β-Bourbonene	-	
Elemol	-	
β-caryophyllene	0.3	
β-Selinene	-	
α-Humulene	0.3	
Allo-aromadendrene	0.5	
γ-muurolene	0.2	
Germacrene D	2.3	
γ-Cadinene	0.1	
α-Muurolene	0.2	
Caryophyllene oxide	0.1	
Cedrol	8.3	
β-Eudesmol	0.2	
α-Eudesmol	0.3	
Mannoyl oxide	-

**Table 3:** Showing the pharmacological properties of *Cupressus sempervirens*

Pharmacological effects	Experimental approach	Key findings	References
Anti-microbial	In vitro tests	Antibacterial and anti-fungi essential oil action	Zhang, et al., 2012; Emami, et al. 2009
Anti-viral	Under a light magnifying lens utilizing the hematoxylin and eosin technique	Anti-HSV action	Amouroux et al. 1998
Anti-parasitic	Centrifugal slim layer chromatography	Action against third instar larva of the mosquito *Culex pipiens*	Moussa et al. 2011
Anti-oxidant	In vitro and in vivo	Paracetamol-induced hepatotoxicity rat model	Loizzo, Tundis et al. 2008
Anti-cancer	Rat model prostatic hyperplasia	Nephridial adenocarcinoma cells and C32 amelanotic melanoma Antiproliferative action against BPH stroma cells in humans	Verma et al. 2014 Donya and Ibrahim 2012
Hypolipidemic	Lipid profile	Substantial decrease in serum fatty oils	Ali et al. 2010
Insecticidal	Centrifugal slim layer chromatography	Antileishmanial action Delayed lethal effect in pupae and grown-ups after larval treatment	Tumen et al. 2012 Moussa et al. 2011
Hepatoprotective	Rat model	Hepatoprotective	Demetzos et al. 2003
Osteogenic	Alkaline phosphatase, and mineralization assay also osteogenic genes expression, osteoblast transcription factor and bone morphogenetic protein 2, in primary cultures of the calvarium extracted from newborn mice	Osteoprotective effects	Ulusal et al. 2006
Wound healing/anti-coagulant	Linear incision and circular excision wound models	Limited wound healing abilities Anticoagulant properties	(Graziani et al. 1997)
Anticholinesterase	Acetylcholinesterase (AChE), tyrosinase (TYRO) and butyrylcholinesterase (BuChE)	Anti-acetylcholinesterase effect	Khan et al. 2014)

(Hassanzadeh Khayyat et al. 2005).

**Table:** 

Phytochemicals	Leaves	Branches	Cones
1.9% saponins			
0.7% alkaloids			
0.31% tannins			
0.22% flavonoids			
0.067% phenols			
Sesquiterpenes	21.9%	14.9%	18.26%
Monoterpenes	42.7% and 43.21	60.4%	
Diterpenes			

Essential oil constituent of two species of *C. sempervirens*

**Table:** 

Mediterranean cypress oil (*Cupressus sempervirens)*	Tunisian *Cupressus sempervirens*	Tunisian *Cupressus sempervirens* continued	Tunisian *Cupressus sempervirens* continued
Tricycle	α-pinene	β-bisabolene	α-campholenal
α-thuyen	δ-3-carene	Cubebol	Camphre
β-pinene	α-thujene	Cis-calmanene	Borneol
Camphor	Tricycle	δ-cadinene	δ-terpineol
Myrcene	α-fenchene	α-copan-11-ol	Myrtenal
δ-3-carene carvacrol	α-pinene	α-calacoreno	Myrtenol
p-cymene	Sabinene	Elemol	Terpen-4-ol
α-pinene	β-pinene	Germacrene B	α-terpineol
Camphene	β-myrcene	β-calacoreno	Isobornyl acetate
Sabinene	α-phelandrene	Caryophyllene oxide	α-terpenyl acetate
Bronyl acetate	δ-3-carene	α-cedrol	Longifolene
β-caryophyllene	Cineol	T-cadinol	Caryophyllene
Germacrene-D	p-cymene	T-murrolol	α-humulene
δ-cadinene-limonene	Limonene	Mannyl oxide	Ermacrene D
γ-terpinene	β-phelandrene	Abiethatriene	β-selene
α-terpinolene	α-terpinolene	Abiethadiene	α-murrolene
α-cedrole	Linalool	Nezukol	Epi-zonarene
	Tartarol	Sempervirol	

## Method of essential oil extraction and quantification and the chemicals structure of the essential oils

*Cupressus sempervirens* cv cereiformis growing in Iran

**Table:** 

Essential oil phytochemical	Leaves (%)	Fruits (%)	Cones (%)	References
α-pinene	39.0	30.0	-	(Miloš, Mastelić et al. 1998)
Sabinene	3.0	2.0	-	(Miloš, Mastelić et al. 1998)
β-pinene	2.2	2.6	-	(Miloš, Mastelić et al. 1998)
Myrcene	3.9	4.1	-	(Miloš, Mastelić et al. 1998)
Δ -3-carene	24.0	24.0	-	(Miloš, Mastelić et al. 1998)
Limonene	3.0	4.0	-	(Miloš, Mastelić et al. 1998)
Terpinolene	4.3	6.6	-	(Miloš, Mastelić et al. 1998)
Bronyl acetate	Traces	1.7	-	(Miloš, Mastelić et al. 1998)
α-terpenyl acetate	6.6	5.6	-	(Miloš, Mastelić et al. 1998)
β-caryophyllene	1.2	Traces	-	(Miloš, Mastelić et al. 1998)
α-humulene	1.3	Traces	-	(Miloš, Mastelić et al. 1998)
Germacrene D	1.7	4.0	-	(Miloš, Mastelić et al. 1998)
Monoterpenic hydrocarbons	79.4	73.3	-	(Miloš, Mastelić et al. 1998)
Monerpenes containing oxygen	7.3	6.6	-	(Miloš, Mastelić et al. 1998)
Sesquiterpenic hydrocarbons	1.7	10.5	-	(Miloš, Mastelić et al. 1998)
Sesquiterpenes oxygen	4.0	Trace	-	(Miloš, Mastelić et al. 1998)
5-isopropyl-2-methyl-1,4-benzoquinone	-	-	3.7–9.7	Krishnaveni, Amsavalli et al. 2013
Hydroxybenzoic acid methyl ester	-	-	15.5	Krishnaveni, Amsavalli et al. 2013
p-cymene-8-ol and	-	-	5.3–6.4	Krishnaveni, Amsavalli et al. 2013
Perilla alcohol	-	-	3.6–8.2	Krishnaveni, Amsavalli et al. 2013
Carvacrol	-	-	2.5–6.3	
2-phenylethanol	-	-	2.7–6.9	
6-deoxytaxodione	-	-	+	(Tumen, Senol et al. 2012)
Ferruginol	-	-	+	(Tumen, Senol et al. 2012)
Taxodione	-	-	+	(Tumen, Senol et al. 2012)
Sugiol	-	-	+	(Tumen, Senol et al. 2012)
Transcommunic acid	-	-	+	(Tumen, Senol et al. 2012)
Imbricatolic acid	-	-	+	(Tumen, Senol et al. 2012)
15-acetoxy imbricatolic acid	-	-	+	(Tumen, Senol et al. 2012)
Flavonoids	+	-	-	

From the adherent leaves of *Cupressus sempervirens* cv cereiformis growing in Iran, essential oils were obtained from fresh fruits and terminal twigs that were fractionated by GC–MS. *Cupressus sempervirens* can be categorized under *medicinal* and aromatic plants (MAPs). These plants are directly used or can be processed by hydrodistillation (HD) to obtain essential oils or by extraction to obtain what must be termed ‘volatile fractions’. These oils are used in pharmacy, cosmetics, perfumes and food industry among others. The compounds identified in essential oils are thirteen in number. Mainly, the components of both the fruits and the leaves were -3-carene, α-pinene, terpinolene and α-terpenyl acetate, although the identified volatile oil is from *Cupressus sempervirens L* cv cereiformis. Leaves and fruit% respectively were: α-pinene 39.0 and 30.0, sabinene 3.0 and 2.0, β-pinene 2.2 and 2.6, myrcene 3.9 and 4.1, Δ-3-carene 24.0 and 24.0, limonene 3.0 and 4.0, terpinolene 4.3 and 6.6, traces and 1.7 of bronyl acetate, α-terpenyl acetate 6.6 and 5.6, 1.2 and traces of β-caryophyllene, 1.3 and traces of α-humulene, germacrene D 1.7 and 4.0, while grouped compounds: (monoterpenic hydrocarbons 79.4, 73.3); (monerpenes containing oxygen 7.3 and 6.6); (sesquiterpenic hydrocarbons 1.7 and 10.5); (sesquiterpenes containing 4.0 and trace oxygen) (Miloš et al. 1998). Using hot and cold ethyl acetate extraction, extraction of glycosides from cones of fresh cypress, *Cupressus sempervirens*, was carried out. Eighteen aglycones were released from enzymatic hydrolysis using β-glucosidase. The glucosidically bound volatile compounds represented 7–8 mg/kg. The major aglycones included 3 thymoquinone (5-isopropyl-2-methyl-1,4-benzoquinone: 3.7–9.7%) and -hydroxybenzoic acid methyl ester (15.5%). Other important aglycones were p-cymene-8-ol (5.3–6.4%) and perilla alcohol (3.6–8.2%), and carvacrol (2.5–6.3%) and 2-phenylethanol (2.7–6.9%). Aglycones with glycosidic bonds and simultaneously free compounds found in essential oil were found to be different (Krishnaveni et al. 2013). 6-Deoxytaxodione (11-hydroxy-7, 9 (11), diterpenes, 13-abietatrien-12-one), ferruginol, taxodione, sugiol, transcommunic acid, imbricatolic acid and 15-acetoxy imbricatolic acid were isolated from *Cupressus sempervirens* (Tumen et al. 2012). The total of flavonoids was 9.5 (mg of quercetin/g of extract), and the total phenolic content of the fresh leaves of *Cupressus sempervirens* was 4.35 (mg of gallic acid/g of extract) (Shahid et al. 2013).

This cypress oil was extracted by using the hydro-distillation method, using a clevenger apparatus. *Cupressus sempervirens* leaves were collected from Hit city in Al-Anbar province—Iraq (Hamid et al. 2021). The influences of three important parameters on the process of oil extraction, water which is used as a solvent to the solid ratio (5:1 and 14:1 (ml solvent/g plant)), temperature (30 to 100 °C) and processing time, were examined to obtain the best processing conditions to achieve the maximum yield of the essential oil. Therefore, the best conditions, which were obtained from a study, were at 4 h as a reaction time, the temperature of about 100 °C and solvent to solid ratios of 10:1 ml solvent/g plant (Hamid et al. 2021). The volumetric mass transfer coefficient at ratios 10:1 and 14:1 solvent to solid ratio were 0.017 and 0.007 min respectively. Thus, it took less time to extract the *Cupressus sempervirens* oil by using the 10:1 compared to using 14:1 solvent to solid ratio. The composition compounds of extracted *C. sempervirens L.* essential oil were analyzed and identified by gas chromatography mass spectrometry (GC–MS) (Hamid et al. 2021).

Comparison between the main compounds of *C. sempervirens* L. leaves essential oil in different countries (origins) (Hamid et al. 2021).

**Table:** 

––––––––a-pinene	3-carene	a-cedrol	a-terpinylacetate	b-myrcene	Germacrene D	Reference
–	15.40	22.64	2.68	$-$	2.91	
54.10	10.80	4.90	5.5	4.6	2.40	[18]
37.10	19.60	1.69	$-$	3.60	1.30	[19]
27.50	13.2	19.30	0.20	1.00	12.10	[20]
36.50	22.17	$-$	4.76	3.16	12.81	[21]
48.60	22.10	3.50	–	4.10	1.60	[22]
6.90	17.85	21.29	–	–	2.75	[23]
30.00	24.00	–	6.60	4.10	4.00	[24]

## Traditional use

**Fig. 2:**
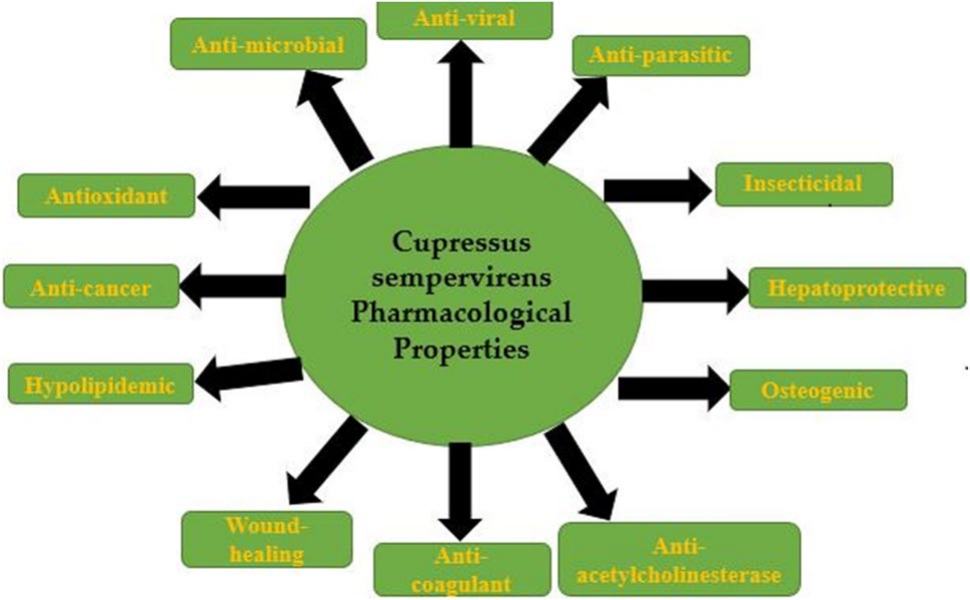
Schematic diagram for the pharmacological properties

The drug was used orally for coughs, colds and bronchitis. (Al-Snafi 2018). A decoction of *Cupressus sempervirens leaves* and cones was used in a sitz bath three times a day for 1 week for hemorrhoids. The cones and leaves were used orally for internal illnesses. Externally, cypress extract was combined to give formulations (ointments and suppositories) and was used in the treatment of hemorrhoids, venous circulation disorders and varicose veins. The essential oil was utilized as an antispasmodic for persistent coughs and antiseptic (Rawat et al. 2010). Cypress has also been used as diuretic to increase kidney’s and venous circulation, improve bladder tone and treat urinary incontinence and enuresis (Mahmood et al. 2013). The parts of the plant utilized for medicinal purposes were the cones and leaves (Rawat et al. 2010) (Fig. 2).

## Pharmacological effects

### Antifungal effects and antibacterial

The assessment of ethanol’s antibacterial action, extracts of ethyl acetic acid and methanol from the aerial parts of *Cupressus sempervirens* against different plants including *Klebsiella pneumonia* (MTCC618*), Staphylococcus aureus* (ATCC6538), *Bacillus subtilis* (ATCC6633), *Escherichia coli* (ATCC15224), *Pseudomonas aeruginosa* (ATCC6643) and *Salmonella typhimurium* (ATCC13048). These extracts’ concentration was utilized (1, 2, 3, 5, 7.5, 10, 12.5 and 15 mg/ml). All *Cupressus sempervirens* extract administered showed a dose-dependent inhibition of bacterial development against all microbes examined (Boukhris et al. 2012) which were six bacterial strains *Proteus vulgaris, Bacillus subtilis, Salmonella typhi* (Gram-negative), *Staphylococcus aureus* (Gram-positive), *Escherichia coli, Pseudomonas aeruginosa* and parasitic species of *Aspergillus niger and Candida albicans*. *Cupressus sempervirens* was used to assess the bactericidal and antifungal action of *Cupressus sempervirens* chloroform and water extracts’ high intensity against Gram positive microbes (inhibition zone of 9–12 mm and 9–14 mm and chloroform and water extracts respectively) and reduced action against Gram negative microorganisms (zone of inhibition 1–6 mm and 1–5 mm water extract and chloroform extract). In any case, the water extract did not show much potency against growths; however, the chloroform extract displayed slight action against *Candida albicans* (3 mm) (Hassanzadeh Khayyat et al. 2005). *Cupressus sempervirens leaves* bactericidal activity of ethanolic, ethyl acetic acid and methanolic extract was accessed using agar well diffusion technique against six bacteria (*Bacillus subtilis, Escherichia coli, Staphylococcus aureus, Salmoniae and Klebsiella pneumonia, Pseudomonas aeruginosa*). Methanolic extract shows huge antimicrobial activities followed by the ethyl acetate and ethanol extracts. The methanolic extract demonstrated greatest inhibitory activity against *B. subtilis, K. pneumonia and S. aureus*. The ethanolic extract showed its action against *P. aeruginosa*, while the ethyl acetic acid extract of *Cupressus sempervirens* demonstrated a more prominent inhibitory action against *E. coli and S. typhimurium* (Zhang et al. 2012). The antimicrobial activities of essential oil were against Gram positive bacteria and other bacteria like (*Bacillus cereus, Staphylococcus aureus, Enterococcus \feacalis, Serratia marcescens*) and Gram negative (*Proteosa vulgarommonis aeruginella, Escherichia coli, Klebsiella pneumonia indica, Aeromonas hydrophila*); the inhibitory zones have diameter of 4 to 12 mm, with MBC and MIC values ​​ranging somewhere in the range of 62.5 and 250 μg/ml. In spite of the fact that *Cupressus sempervirens* methanolic extract firmly restricted the progression of many bacteria growth (Ismail et al. 2013a). Studies were carried out to find out the antimicrobial action of *Cupressus sempervirens* essential oil against ten fungi and bacteria (*Bacillus subtilis, Escherichia coli, Pseudomonas aeruginosa, Staphylococcus aureus Halomonas prolong, Aspergillus alma, Salmonella typhimurium, Enterococcus hirae*). The outcome revealed that *Cupressus sempervirens* oil restricted the bacteria development, yeast and filamentous growths. The MCC and MIC values ​​showed that the *Cupressus sempervirens* essential oil remained exceptionally powerful. Therefore, the MIC/MCC proportion affirmed antibacterial and anti-fungi essential oil action. In spite of the fact that the antimicrobial action of *Cupressus sempervirens* essential oils was evident against Gram-positive compared to Gram-negative bacteria (Toroglu, 2007). The inhibition zones are 2 and 4 µl/circle of *Cupressus sempervirens* essential oil against the microorganisms examined ( separately): *Staphylococcus aureus 7 and 8; Micrococcus luteus 10 and 13; Mycobacterium simegmatis* 10 and 11; *Bacillus megaterium* 7 and 9; *Enterococcus faecalis* 7 and 9; *Streptococcus faecalis* 7 and 9; *Saccharomyces cerevisiae 9 and 10; Bacillus brevis 7 and 8; Pseudomonas pyocyaneus 9 and 11; Aeoromonas hydrophila 7 and 10; Yersinia enterolitica 8 and 9; and Klyveromyces fragilis* 15 and 17 mm (Amri et al. 2013). *Cupressus sempervirens* essential oil was inspected in three microscopic organisms (*Bifidobacterium lactis, Micrococcus luteus and Escherichia coli*) and seven parasites (*Aspergillus flavous, Aspergillus niger, Aspergillus fumigatus, Fusarium oxysporium, Fusarium solani, Camposipterus* and *Penicillium digitatum*). Essential oil inhibition zones for 96 h of brooding against *Bifidobacterium lactis* were 24.05 mm, *Escherichia coli* was 16.11 mm, while *Micrococcus luteus* was 11.90 mm. Concerning essential oil fungicidal action inhibition zones were 5.7 mm against *F. solani* and 29 mm against *P. digitatum* following 96 h of incubation (Al-Othman et al. 2012). Compounds, like, diterpenes, 13-abietatrien-12-one, 6-deoxytaxodione (11-hydroxy-7, 9 (11) taxodione) separated from the cones (products) of Cupressus *sempervirens* indicated high bactericidal action (IC50 is 0.80 and 0.85 μg/ml) against methicillin-resistant *Staphylococcus aureus* (Tumen et al. 2012). The antifungal action of Cupressus *sempervirens* essential oil tried against 8 organisms from cultured media was analyzed in an in vitro test (*Fusarium culmorum, Fusarium oxysporum, Fusarium equisiti, Fusarium verticillioides, Fusarium nygamai, Botrytis cinerea, var. Nivale var. Alternaria sp and Microdochium nivale*), and all *Cupressus sempervirens* essential oil tests demonstrated astounding antifungal control against all growths examined (Hassanzadeh Khayyat et al. 2005). The *Cupressus sempervirens* var. Dupreziana leaves essential oils were analyzed for antifungal activities against 10 types of agricultural fungi (*Fusarium culmorum, Gibberella avenacea, Microdochium nivale, Fusarium oxysporum, Fusarium verticillioides, Fusarium subglutinans, Rhizoctonia solani, Alternaria culmorum, Fusarium nygamai* and *Fusarium alternamorum*). Antifungal in vitro tests were tried on the oils, and the effect revealed a serious growth inhibition of 10 plant pathogenic fungi (Emami et al. 2009).

### Antiviral effect

Earlier investigations have been steered to assess the influence on herpes infections (HSV-1) that are treated with *Cupressus sempervirens* ethanolic extracts of *Cupressus sempervirens* var. cereiformis and *C. sempervirens* var. horizontalis. Herpes infection (HSV-1) contamination was performed on HeLa cell monolayers to assess the antiviral property of plant extract under a light magnifying lens utilizing the hematoxylin and eosin technique. Test outcomes were estimated against acyclovir, a positive control. As indicated by the outcomes, antiviral properties against the HSV-1 infection were seen in the three plants. The strongest extract was that of *C. sempervirens*. Of the different parts tried, the organic product extract was appeared to have the highest anti-HSV action (92). The in vitro assessment done indicated that a small amount of proanthocyanidin polymer (MW 1500–2000 Daltons) was extracted from *Cupressus sempervirens L.* (Amouroux et al. 1998).

### Antiparasitic and insecticidal effect

Antiparasitic activities of the ethanolic extract of *Cupressus sempervirens* powder cones, gathered in Oxford, Mississippi, were examined. The utilization of centrifugal slim layer chromatography caused the isolation of many 13-abietatrien-12-one), 6-deoxytaxodione (11-hydroxy-7,9 (11), diterpenes, taxodione, sugiol and ferruginol. 6-Deoxytaxodione (11-hydroxy-7, 9 (11), 13-abietatrien-12-one) of which taxodione indicated a solid antileishmanial action with values ​​of a fraction—greatest inhibitory focus (IC50) of 0.077 μg/ml and 0.025 μg/ml, separately, against promastigotes *of Leishmania donovani,* compared with those of the standard antileishmanial drugs, pentamidine (IC50 1.62 μg/ml) and amphotericin B (IC50 0.11 μg/ml) (Tumen et al. 2012). Ethanol, acetone and crude oil ether extracts from Egyptian *Cupressus sempervirens leaves* action against third instar larva of the mosquito *Culex pipiens* were assessed. The outcome demonstrated that crude oil ether extract was more effective than the acetone and ethanolic extract, in this way indicating their antiparasitic property. The toxicity examined, in light of the LC50 esteems, were: ethanolic (LC50 263.6 ppm) > acetone extract (LC50 104.3 ppm) > crude oil ether separates (LC50 37.8 ppm). A huge decrease was seen in both the level of pupae and the growth of the adults although all the extracts demonstrated a delayed lethal effect in pupae and grown-ups after larval treatment. Furthermore, diverse degrees of morphogenic deviations from the norm were seen in the young and adult stages (Moussa et al. 2011).

### Antioxidant effect

The methanol and chloroform extracts from *Cupressus sempervirens leaf* were examined to ascertain antioxidant action utilizing the DPPH assay. Methanol extract action (50 μg/ml) against radicals was 65% unlike the chloroform extract (50 μg/ml) which is 6% (Asgary et al. 2013). The antioxidant action of fresh leaves of *Cupressus sempervirens* by checking test of nitric oxide was 1.17 (mg quercetin/g of extract), reducing power test was 2.85 (mg of ascorbic acid/g of extract); via metal chelation the antioxidant action of two varieties of extracts was tried utilizing 2,2-diphenyl-1-picrylhydrazyl (DPPH) and N, N-dimethyl-p-phenylenediamine (DMPD) radical scavenging action, chelating capacity of metals along with ferric-(FRAP) and lessening antioxidant intensity of phosphorus-molybdenum (PRAP). The antioxidant activities test was done at 2000 µg/ml. From the exploration discovering, there was variety in antioxidant activities because of the technique utilized. For example, *Cupressus sempervirens* ethyl acetate cone extract var. *horizontalis* indicated greatest DPPH radical scavenging activities (87.53 ± 0.17%), while just six of the extracts had the capacity to displace the radical of DMPD with a range of 6.06 ± 0.23 and 30.34 ± 0 69%. The resultant effects of the FRAP test show that *Cupressus sempervirens* cone acetone extract var. horizontalis demonstrated the absorbance value at its peak showing elevated antioxidant action, despite that the extracts commonly had low action in the PRAP examined. Methanolic extract of the leaf of *Cupressus sempervirens* var. horizontalis has the elevated antioxidant action. The cone and leaf methanol extract lack metal chelating limit in the metal chelation test. Conversely, *Cupressus sempervirens* ethyl acetate foliar extracts var. horizontalis (75.86 ± 0.33%) and *Cupressus sempervirens* var. pyramidalis (77.07 ± 3.22%) indicated the maximum activity in this test. *Cupressus sempervirens* essential oil was examined for its antioxidant property by estimating the scavenging effect of the radical on 2,2-diphenyl-1-picrylhydrazyl (DPPH) and utilizing 2,2′-azinobis (3-ethylbenzothiazoline-6-sulfonate) (ABTS) paper. The test result showed elevated antioxidant activities (2.14 mM and 7.7 μg/ml and Trolox for the ABTS and DPPH tests, separately) matched with BHT (Toroglu 2007). After extraction of the essential oils, assessment of the antioxidant and antagonistic effect to glycation action of *Cupressus sempervirens* var. *horizontalis* branch and fruit oils was done. *Cupressus sempervirens var. horizontalis* twigs and other product were accessed for essential oils isolation by the use of steam refining technique. A gas chromatography–mass spectrometry technique was utilized to assess the quantity of essential oils. While trying to assess the antioxidant effects of oils at various fixations (180, 220 and 260 μg/ml), the peroxyl radical-interceded red platelet hemolysis (RBC) and linoleic acid peroxidation test and test were utilized. The peroxidation of linoleic acid for 4 h was assessed every incubation hour. The anti-glycation action of the oils at 600, 400, 200 µg/ml was observed utilizing glycation surveys for hemoglobin and insulin. Hemoglobin glycation was restrained both by the twigs (54, 62.6 and 44.8%) and by the natural products (48.5, 62.8, 41.0%) at 600, 400, 200 μg/ml of oil respectively. The inhibition rates of insulin glycation include (73.8, 69.2, 66.1%) that of twig oil and (81.5, 76.9, 80.0%) that of oil of natural products at 600, 400, 200 μg/ml, respectively. The red platelet hemolysis was further inhibited by twig oil (15.0, 38.5, 49.9%) and natural product oil (25, 38.6, 45.9%) at 260, 220, 180 μg/ml, respectively. Lastly, there is decreased linoleic acid peroxidation by the oils which arrived at its greatest point after 4 h for the two twigs (53.4, 35.6, 39.5%) and natural products (59.8, 58.6, 47.5%) at 260, 220, 180 μg/ml of oil separately; 600 and 400 µg/ml were assessed utilizing insulin and hemoglobin glycation surveys. The glycation of hemoglobin was inhibited both by the twigs (44.8, 62.6 and 54.0%) and by the organic products and (48.5, 62.8, 41.0%) at 600, 400 and 200 μg/ml of oil respectively; the inhibition rates of insulin glycation were (73.8, 69.2, 66.1%) for twig oil and (81.5, 76.9, 80.0%) for organic product oil 600, 400 and 200 μg/ml, respectively. The twig oil inhibited red platelets hemolysis by (15.0, 38.5 and 49.9%) including natural product oil (25.0, 38.6 and 45.9%) at 260, 220, 180 μg/ml, respectively. To conclude, linoleic acid peroxidation was lessened by the oil; after 4 h the greatest point was reached for the two twigs (53.4, 35.6 and 39.5%) and also for organic products (59.8, 58.6 and 47.5%) at 260, 220 and 180 μg/ml of oil individually. (Ali et al. 2012). The antioxidant properties of *Cupressus sempervirens leaves* hydroethanolic extract were evaluated in vitro, and comparison was made with ascorbic acid and also the hepatoprotective activity in vivo in a paracetamol-induced hepatotoxicity rat model. From the in vitro study, the extract was shown to contain high volume of phenolic compounds including flavonoids; this can be linked to the antioxidant potential in various rat models. The study was done in vivo for 4 weeks; pretreatments with silymarin (100 mg/kg/day, po) or extract (250 mg/kg/day, po) or) show the safety index in normal rats and hepatoprotective action against toxicity of paracetamol (4 g/kg bw, po) (Loizzo et al. 2008).

### Anticancer effect

Various compounds from plants have been shown to have anti-cancer properties either on cell line model or rat model with great anti-proliferative potential (Teibo et al. 2021a, b). With the assistance of the sulforhodamine B examined, the essential oil of *Cupressus sempervirens* sp. Pyramidalis, the antiproliferative action of was inspected in nephridial adenocarcinoma cells and C32 amelanotic melanoma cells. The leaf oil of *Cupressus sempervirens* ssp. Pyramidalis showed the maximum cytotoxic action with an IC50 estimation of 104.90 microg/ml against C32 (Verma et al. 2014). The proliferation of BPH stromal cells in humans was inhibited by the *Cupressus sempervirens* (CS) ethanolic extract of the fruits of the soil activity given in its part rich in diterpene, solvent in chloroform. Eight essential diterpenes were gotten indicating mild to strong action with the strongest diterpene (labda-8 (17), 12,14-trien-19-oic acid) showed an IC50 of 37.5 μM (with antiproliferative action against BPH stroma cells in humans). There was a significant inhibition of Stat-3 enactment (phosphorylation) in BPH stromal cells and inhibited androgen-delicate KLK3/PSA transamination in LNCaP cells TMPRSS2 qualities. The CS fraction in acid12, 14-trien-19-oico, labda-8 (17), inhibited rat model prostatic hyperplasia thereby causing TUNEL marking in stromal cells with lower articulations of bcl portion—2/bax, IGF-I, TGF-ß and PCNA (Donya and Ibrahim 2012). A strong cytotoxic activity of taxodione disengaged from *Cupressus sempervirens* cones (natural products) was monitored. Utilizing the trial rat model, paracetamol was utilized to induce liver toxicity in rats, *Cupressus sempervirens* hydroethanolic extract were used to find out the hepatoprotective and anticancer activities of while using silymarin as reference agent. A 4-week study from previous researches has used silymarin (50 mg/kg/day, po) or hydroethanolic extract (250 mg/kg/day, po) or) showing hepatoprotective activities compared to one toxic dose of paracetamol (4 g/kg bw) and a good profile in normal rats (Demetzos et al. 2003).

### Hypolipidemic effect

Research on the lipid profile of the cone extract effect of *Cupressus sempervirens* (CSE) in Wistar rats was done with hypolipidemic effect. The extract orally administered caused a huge reduction in total serum cholesterol, which remain significant for a period of 6 weeks. After the beginning of treatment (*p* < 0.001), the animals were presented with lower cholesterol levels at (*p* < 0.05); there was a substantial decrease in serum fatty oils, contrasting 0 weeks and 6–24 weeks. It was seen that between the animals treated with CSE and the control there were no significant difference in fatty substance levels and HDL cholesterol level throughout the experiment (Ali et al. 2010).

### Protective effect

Biochemical assays like total serum protein, creatinine, albumin, urea, LDH tests and histopathological assessments were performed to assess the curative effect against CCl_4_ hepatotoxicity utilizing *Cupressus sempervirens* extract. In female Wistar rat, one intraperitoneal administration of 10% CCl_4_ was done in olive oil (1 ml/kg body weight) (positive control), another group got CCl_4_ and were treated with *Cupressus sempervirens* extract thrice each week for 6 weeks and a group got CCl_4_ for about 6 weeks and were allowed to recover by themselves. After the experiment, all animals were sacrificed after which biochemical and histological parameters were assayed. There was a clear difference across all the groups. The rats treated with CCl_4_ and left for 6 weeks to recover on their own exhibited mild enhancements in the parameters studied. Administration of plant extract improved the altered biochemical parameters to a large extent. The histopathological evaluation of the liver and kidney of the group treated with *Cupressus sempervirens* was closely related to that of control groups (Koriem 2009). Four-week pretreatment with silymarin (50 mg/kg/day, po) or hydroethanolic extricate (250 mg/kg/day, po) showed good profile in normal rats; also, it indicated hepatoprotective action against the paracetamol toxicity (4 g/kg bw, po) as shown by a rapid decrease in DNA fragmentation and significant reduction in the level of chromosomal distortions in bone marrow cells (Koriem 2009). The assessment of *Cupressus sempervirens* flavonoids protective action (quercetin and rutin) against the toxicological effect of lead acetate on the liver was studied. Thirty male albino rats were distributed into five groups (six rats for each group). The control is group I; Group II got lead acetate of 0.5 mg/g in their feed for 60 days. Group III got 8 mg/100 g body weight of *Cupressus sempervirens* (lyophilized from methanol extract of seeds) 14 days before the administration of lead acetate. Group IV were given 0.3 mg/100 g of body weight of the flavonoid quercetin every day for 14 days before the administration of lead acetate; Group V were given flavonoid daily 0, 1 mg/100 g of body weight for 14 days before lead acetate. Lead acetate was found to cause elevated serum and tissue ALT, ALP, AST and tissue MDA, bilirubin, tissue and plasma NO, regardless of significant elevated serum cholesterol, fatty substances, LDL and HDL. Though, lead caused a significant reduction in all out serum and tissue protein, albumin/globulin proportion albumin, globulin, SOD and GPx in blood and tissue compared with the benchmark group. Treatment with methanolic extract of *Cupressus sempervirens,* quercetin and rutin for 14 days before lead acetate did not allow increase in parameters. In like manner, treatment with methanolic extract of *Cupressus sempervirens* and its flavonoids can increase the defensive activity against the poisonous effect of lead acetate (Aazza et al. 2011).

### Anti-acetylcholinesterase effect

Assessment of inhibitory action of extracts of ethyl acetate, dichloromethane, acetone and methanol from *Cupressus sempervirens* var. *horizontalis* (CSH) and var. *pyramidalis* (CSP) cones and leaves was performed to counter acetylcholinesterase (AChE), tyrosinase (TYRO) and butyrylcholinesterase (BuChE). The extracts demonstrated mild to adequate cholinesterase inhibition at 200 µg/ml. The CSP cone dichloromethane extricate demonstrated the maximum inhibition (36.10 ± 1.45%) against AChE, while the greatest inhibition (40.01 ± 0.77%) against BuChE was done by the CSH leaf acetone extract (Fleming 2000). The *Cupressus sempervirens* essential oil was investigated as anti-acetylcholinesterase. It demonstrated that the inhibitory group of essential oil (IC50) was 0.2837 ± 0.0115 mg/ml (Khan et al. 2014).

### Osteogenic effect

The validated model for osteogenic effect was assessed; this includes the alkaline phosphatase, and mineralization assay, osteogenic genes expression, osteoblast transcription factor and bone morphogenetic protein 2, in primary cultures of the calvarium extracted from newborn mice using four diterpenoids (sugiol, 15-acetoxy imbricatolic acid, transcommunic acid and imbricatolic acid). Among the four studied, it was observed that the dose of 1.0 mg/kg bw of sugiol showed osteoprotective effects significantly, but at the same dose there was not uterine estrogenicity. Additionally, there was an improvement of biomechanical properties, as demonstrated by increase energy, power and rigidity in the femoral bones in the group treated with sugiol compared to the Ovx animals that were untreated (Ulusal et al. 2006).

### Anticoagulant and effect on the viability of isochemically challenged folds

Cone water extract pretreated with cypress prolonged the blend of endothelial-determined nitric oxide (eNO) in rats from endothelial cellular function in isolated aortic rings assessed in vitro. Also, it had anticoagulant properties. Because of these effects, their effect on the endurance of arbitrary augmentations of ischemic pivotal folds was analyzed. The previously treated group was treated for 7 days with 30% cypress cone water extract before a fold rise, another 3 days a short time later. The ischemic objective was a 6 × 7 cm islet epigastric supply route flap dependent on the inferior epigastric pedicle at the right. At the time of the conclusion of the research, hemodynamic factors, mean arterial pressure including the pulse rate were evaluated. Flap endurance and perfusion rates were assessed using laser Doppler flowmetry and microangiography. The aortic isometric pressure separated from the control and pretreated groups was extracted to mirror the vascular feedback. Dose–response connections to acetylcholine were resolved and placed side by side with the control group. Hemodynamic factors did not show any difference. In the group pretreated, micro angiograms caused prolonged angiogenesis, slender thickness and expanded folds’ perfusion (blood perfusion parts) in the proximal and distal parts (*p* < 0.05). The endothelium-derived nitric oxide-associated maximal unwinding (Emax) and EC50 approved for acetylcholine were notably higher in the pretreated group compared with the control. From the information, it was proposed isochemically tested rats pretreated with cypress water extracts build the suitability (Tumen et al. 2012).

### Wound healing effect

The healing and mitigating effects of wounds were assessed from essential oils got from *Cupressus* cones. The linear incision and circular excision wound models were used to evaluate wound healing in vivo using hydroxyproline content assessment, then histopathological research. The regenerative potential was evaluated with Madecassol balm as control or standard. Likewise, the anti-inflammatory effect of oil was checked using acetic acid-induced capillary permeability test. *Cupressus sempervirens* var. *horizontalis* and *Cupressus sempervirens* var. *pyramidalis essential* oils did not bring about serious healing (Graziani et al. 1997).

## Pharmacokinetic studies

### Amentoflavone

In assessment done on the pharmacokinetic, administration of amentoflavone to rats was intraperitoneally (ip, 10 mg/kg, intravenously (iv, 10 mg/kg) and orally (po, 300 mg/kg)). From the outcome, it was discovered that 90.7% of the total amentoflavone circulated as metabolite that are conjugated after oral administration. The infusion ip and iv in the plasma of rats 70.2% ± 5.18% and 73.2% ± 6.29% became metabolites from amentoflavone administered altogether. Moreover, the compound bioavailability declined in the ip infusion (77.4% ± 28.0%) than oral intake 0.04% ± 0.01% (Liao et al. 2015). The pharmacokinetics of amentoflavone alone or along with different constituent have been studied in hyperlipidemic and normal rats’ models (Liao et al. 2015). Bioflavonoid administered orally has amentoflavone T1/2 and Tmax as 2.06 h ± 0.13 h, 1.13 h ± 0.44 h in normal rats and 1.91 h ± 0.32 h, 0.96 h ± 0.10 h in model rats, respectively. A renowned Chinese drug is Shixiao San that contains amentoflavone (Zhou et al. 2013). Subsequent to the oral administration of a Shixiao San medication, amenoflavone T1/2 and Tmax in the normal rat and rat model were 3.34 h ± 0.37 h, 4.00 h ± 0.00 h and 4.19 h ± 0.64 h, 4.17 h ± 0.40 h respectively.

Recently, pharmacokinetic investigations of extracts and bioactive combinations from TCM and characteristic medication have become research patterns. Amentoflavone was not special in anyway, as a delegate bioflavonoid with a few pharmacological capabilities. The study done on the pharmacokinetics, showed that rats got amentoflavone intraperitoneally (ip, 10 mg/kg), intravenous (iv, 10 mg/kg) and orally (po, 300 mg/kg). Therefore, it was discovered that 90.7% of the total amentoflavone turned into metabolites after oral administration. The iv and ip infusion was seen in rats’ plasma in the placebo; 73.2% _6.29% and 70.2% _ 5.18% of the all amentoflavone were available as formed metabolites (Liao et al. 2015).

### Monoterpenes

From research by Cheng et al. (2016) the paeoniflorin bioavailability in a single ‘four-site’ rat intestinal perfusion model and refined Caco-2 cells was seen orally. The retention was slower than the aglycone (paeoniflorigenin) in cultured cells. It was seen that some factor pointing out the poor bioavailability of paeoniflorin was because of helpless saturation, efflux mediated by P-gp and hydrolysis through glucosidase. At the point of *Paonia lactiflora* (Chishao) crude extraction from roots as the major component (85.5%) was given to human subjects intravenously and through multiple infusion; the half-lives were − 1.2–1.3 h for paeoniflorin. Following the exposure to major organs (Cheng et al. 2016) the bioavailability of paeoniflorin has been seen to be lower in rabbits (7.24%) and rats (3.24%) after oral administration (Martey et al. 2013). Monoterpene agicones like thymol and carvacrol have moderate retention in circulation system after oral administration (Austgulen et al. 1987). Unchanged glucuronide and sulfate forms have been found, too. As Dong et al. showed that cavacrol, uridine-diphosphate glucuronyl transferase is a substrate, that can repress the catalyst (Dong et al. 2012).

In research conducted to assess the possible anticancer properties, limonene was given to women with recently analyzed operable malignancy in the tissue of the breast, arriving at a high tissue focus (mean 41.3 μg/g of tissue) while its essential urging dynamic metabolite was accounted for peric acid (Miller et al. 2010). Past studies done by Li et al. (2012) have demonstrated the concentration of limonene in fat tissue (TA) (Li et al. 2012). Possible digestion and/or discharge of swertiamarin is liver and kidney from oral administration in rats confirming fast and wide movement in tissues with the greatest amount (Cheng et al. 2014). It was seen that for genopiside, the administration of these combinations as crude plant extract could be desirable for bioavailability over pure compounds (Delea et al. 2003). The function of AMPK in the control of glucose digestion is an objective for DM to which much consideration has been given to lately. Medications that enact AMPK increase phosphorylation of insulin receptor 1 (IRS1) substrate. Phosphorylation of Ser 789 was equal to or greater than the actuation course of phosphoinositide 3 kinase/protein kinase B (PI3K/PKB) signaling (Meng et al. 2015). The notable antidiabetic drug metformin also acts by initiating the AMPK pathway in various target organs (Gaidhu et al. 2009). The anti-obesity effect can be accomplished by actuating AMPK in adipocytes which represses lipogenesis while advancing energy loss (Kim et al. 2016). Even though, a few studies have shown the shocking awareness of glucose activation by AMPK and lipid digestion is essentially identified with the degree of more facts that is expected to set up a reasonable bioavailability and/or pharmacokinetic profile of monoterpene.

Therefore, these combinations can be assimilated/made bioavailable for bioactivity in different organs since they show antidiabetic effects when orally administered in moderate and mild dosages. More studies are needed on the formulations and optimization because of their extreme nonpolar properties, implying their ability to permeate the cell membrane, with its low polarity.

## Toxicity and side effects

After the right administration of the therapeutic dosages, no observable changes were detected. Report by Al-Snafi (2016) indicated that kidney disease can happen with consumption of higher doses. Various researches have demonstrated that *Cupressus sempervirens* pollen was major found in the air during spring winter.

### Cypress essential oil toxicity

In an investigation by Fayed (2015), cypress essential oil was checked if it can be used as anticancer and antioxidant agents. In a 90-day treatment of mice with essential oil of *C. sempervirens*, a few changes were seen in their biochemical parameters, without watching death of the mice during the treatment. In the mice serum albumin, glucose, globulin, fatty oils, uric acid and total protein did not change, while creatinine and total cholesterol diminished essentially (*p* £ 0.05), but urea increased (*p* £ 0.05) compared with the normal control group (Fayed 2015). Serum enzyme experiments done on *C. sempervirens* essential oil showed that the oil caused a critical increase in the transaminase amount (ALT and AST), but the control estimation of ALT (17.12 IU L-1) and AST (25.90 IU L-1), a huge increase in the degrees of 24.05 IU L-1 and 33, 73 IU L-1 of the separate compounds were seen in the essential oil. Besides, a huge elevation in serum LDH action was discovered in mice orally given the essential oil. The serum activity of important phosphatase was not significantly different (*p* £ 0.05) when compared to the control after the oil administration. Actually, all the properties acquired in these groups were within the normal range (Mitruka and Rawnsley 1977). The renal and hepatic tissues serum proteins valuable parameters show a decrease. In this research, no alteration was recorded in the amount of globulin, albumin creatinine, urea and total protein (nitrogenous waste product of metabolism) (Fayed 2015).

The function of renal tissues is obtained by screening for creatinine and urea. Since they remain overseen principally through filtration in the glomerulus with practically zero renal guideline or transformation throughout renal capacity deterioration (Kamal 2014). The massive increase in the concentration of urea in the serum is a pointer to dysfunction of the kidney (Yakubu et al. 2003). The cypress-treated group showed a critical decline in content of creatinine not causing any essential oil toxicity to kidney and muscle (Fayed 2015). Subsequently, the effect of the essential oil of cypress kidney damage investigated recommends toxicity. Serum levels of AST and ALT increase at whenever there is damage to the hepatic cell.

Whenever there is elevation in the enzyme’s activities, there is also serious damage to the liver (Eteng et al. 2009). An elevated serum action of these enzymes in the current research shows that the liver may be affected by the essential oil from cypress. The cell membrane permeability could be affected and penetrable. The circulatory system becomes infused with these enzymes leading to their increase in the serum LDH activity is available in every body cell and is discovered distinctly in the cell cytoplasm. Activities of isozyme are noticed in the erythrocytes, lymph hubs, platelets, leukocytes, kidney, heart, myocardium, lung, liver, skeletal muscle and spleen (Drent et al. 1996). The level of protein in different tissues are seriously elevated compared to serum. In this manner, the levels in the tissue are roughly multiple times greater than the serum, and spillage of the enzymes from damaged tissue mass can elevate LDH level in the serum largely (McMurray 1986). This investigation has demonstrated that cypress oil toxicity is mild. The *Cupressus sempervirens* leaf essential oil is toxic, and it is accounted here for the first time (Fayed 2015).

## Conclusion

From the previous studies, we could infer that *Cupressus sempervirens* extracts has various biological and pharmacological effects. Recent pharmacological discoveries have shown the effects of the extracts in different points of view; these include anti-inflammatory, antioxidant, antidiabetic, antiviral, anti-malignancy and also their effect on cardiovascular and central nervous system. Conversely, these bioactivities originate from animal studies in vivo and in vitro studies though still inconsequential. It is interesting that in vitro bioactivity cannot fully elucidate the biologic effect in vivo, while the animal’s model is essential for pharmacological researches before clinical use. The bioactivities in vitro need to be explored by doing comprehensive research in future studies. Presently, the study done on the pharmacokinetics has recommended that the amentoflavone digestion was fast, and the bioavailability of bioflavonoid after indigestion orally is low in rats. Animal model experiments are few but available. We propose that the bioavailability can be improved by introducing synthetic compounds, modifying them structurally or adding specific drug that meets needs, which might be a focal point of the amentoflavones studies. Nevertheless, since there are some pharmacokinetic contrasts among normal and model animals of the evaluation of Cupressus sempervirens. Overall, the various bio and pharmacological activity of *Cupressus sempervirens* is owned to its rich phytochemicals which can be worked on for drug development in the future.

## Data Availability

Not applicable in this study.
